# Deep Learning Prediction of Response to Anti-VEGF among Diabetic Macular Edema Patients: Treatment Response Analyzer System (TRAS)

**DOI:** 10.3390/diagnostics12020312

**Published:** 2022-01-26

**Authors:** Saif Aldeen Alryalat, Mohammad Al-Antary, Yasmine Arafa, Babak Azad, Cornelia Boldyreff, Tasneem Ghnaimat, Nada Al-Antary, Safa Alfegi, Mutasem Elfalah, Mohammed Abu-Ameerh

**Affiliations:** 1 Department of Ophthalmology, The University of Jordan Hospital, The University of Jordan, Amman 11942, Jordan; mutasemrabie@hotmail.com (M.E.); mohammd_73@yahoo.com (M.A.-A.); 2 School of Computing and Mathematical Sciences, University of Greenwich, London SE10 9LS, UK; M.AlAntary@greenwich.ac.uk (M.A.-A.); Y.Arafa@greenwich.ac.uk (Y.A.); c.boldyreff@greenwich.ac.uk (C.B.); 3 School of Computer Engineering, Iran University of Science and Technology, Tehran 13114-16846, Iran; gbazad93@gmail.com; 4 Department of Computer Science, Princess Sumaya University for Technology, Amman 11941, Jordan; ghnaimat.tasneem@gmail.com; 5 Ibn Al Haytham Hospital, Amman 11190, Jordan; Nada.alantary97@gmail.com; 6 Tripoli Central Hospital, Tripoli 22131, Libya; Safa.Alfegi@gmail.com

**Keywords:** anti-VEGF, artificial intelligence, deep learning, diabetic retinopathy, macular edema

## Abstract

Diabetic macular edema (DME) is the most common cause of visual impairment among patients with diabetes mellitus. Anti-vascular endothelial growth factors (Anti-VEGFs) are considered the first line in its management. The aim of this research has been to develop a deep learning (DL) model for predicting response to intravitreal anti-VEGF injections among DME patients. The research included treatment naive DME patients who were treated with anti-VEGF. Patient’s pre-treatment and post-treatment clinical and macular optical coherence tomography (OCT) were assessed by retina specialists, who annotated pre-treatment images for five prognostic features. Patients were also classified based on their response to treatment in their post-treatment OCT into either good responder, defined as a reduction of thickness by >25% or 50 µm by 3 months, or poor responder. A novel modified U-net DL model for image segmentation, and another DL EfficientNet-B3 model for response classification were developed and implemented for predicting response to anti-VEGF injections among patients with DME. Finally, the classification DL model was compared with different levels of ophthalmology residents and specialists regarding response classification accuracy. The segmentation deep learning model resulted in segmentation accuracy of 95.9%, with a specificity of 98.9%, and a sensitivity of 87.9%. The classification accuracy of classifying patients’ images into good and poor responders reached 75%. Upon comparing the model’s performance with practicing ophthalmology residents, ophthalmologists and retina specialists, the model’s accuracy is comparable to ophthalmologist’s accuracy. The developed DL models can segment and predict response to anti-VEGF treatment among DME patients with comparable accuracy to general ophthalmologists. Further training on a larger dataset is nonetheless needed to yield more accurate response predictions.

## 1. Introduction

Diabetic macular edema (DME) is the most common cause of visual impairment among patients with diabetes mellitus, affecting almost 4% of patients with diabetes [1,2]. Its global prevalence is expected to increase from almost 18.83 million in 2020 to 28.61 million in 2045 where prevalence and increase are expected to be highest in developing countries with limited resources [1]. Currently, anti-vascular endothelial growth factors (Anti-VEGFs) are considered the first line in managing DME [3]. However, those anti-VEGFs carry high costs and burden even in high resource settings [4,5]. Considering the higher prevalence of DME in developing countries, with almost the highest risk among Middle Eastern populations [1], it is important to develop strategies to mitigate this issue and better allocate resources to tackle the problem. The importance of allocating resources has been recently investigated during recent lockdowns, where only a limited number of ophthalmic procedures were allowed in Jordan [6]. In such situations, healthcare providers need to prioritize patients who would better respond to treatment and suffer more adverse outcomes if treatment was delayed. One strategy that has been proposed, but not yet applied sufficiently, is the use of artificial intelligence (AI) and its machine and deep learning derivatives to aid in the prediction of the outcome of anti-VEGF [7].

One of the main problems ophthalmologists typically face when treating DME patients with anti-VEGFs is the unpredictable response to treatment. Most patients respond well to anti-VEGF agents, whereas some might show a moderate or even poor response [8]. 

The importance of predicting the outcome of anti-VEGF from baseline data in low-resource settings might help in triaging the urgency of treatment, providing better patient counseling, and even in selecting appropriate subjects for clinical trials that investigate novel therapies for DME. The aim of this work has been to develop a deep learning model for predicting the response to intravitreal anti-VEGF injections among patients with diabetic macular edema. Prediction is achieved using two connected deep learning models by first segmenting then classifying OCT images acquired before intravitreal anti-VEGF injections. The segmentation model first encodes the local semantic information in the latent space to capture the hidden information for DME segmentation. Then the classification model uses the generated segmentation map, alongside the input image, to predict the effectiveness of anti-VEGF treatment. 

## 2. Methods

### 2.1. Study Participants

This study was approved by the institutional review board of the Jordan University Hospital (IRB approval number 10/2021/17769) and was conducted in concordance with the latest declaration of Helsinki. The study was a collaborative project between The University of Jordan’s Department of Ophthalmology, and the University of Greenwich’s School of Computing and Mathematical Sciences.

The study included patients who had diabetic retinopathy along with DME. DME was defined as central subfield thickness greater than 320 µm for men or 305 µm for women as measured on OCT [9], treatment-naive diabetic macular edema or more than three months since last anti-VEGF injection or more than six months since last steroid injection, and had macular OCT done within 7 days before and 7 days after intravitreal anti-VEGF injections, which were administered within four months period. Patients who had pars plana vitrectomy, undergone intraocular surgeries within the injection period, had ocular diseases or media opacity that affected vision, or patients with poor quality images were excluded from the study. 

The following data were recorded for each patient: data regarding age, gender, severity of diabetic retinopathy through fundus exam, best corrected visual acuity before and after intravitreal anti-VEGF injections, central OCT foveal thickness before and after intravitreal anti-VEGF injections, number of injections given, prior anti-VEGF treatment, phakic status (phakic vs. pseudo-phakic), glycosylated hemoglobin A1c (any reading during the last 3 months), and any other ocular diseases. Data regarding only one eye were included for each patient, in order to avoid in between-eye correlation [10].

### 2.2. OCT Image Acquisition and Preparation

OCT images were extracted from an 8 × 8 mm macular area focused on the fovea captured using the spectral domain OCT (Nevonx, version 7.2.0, OPTOPOL Technology, Zawiercie, Poland). The device’s eye-tracking system compensated for eye movements. The automatic re-scan function using a reference point was activated to minimize variation in allocating the acquisition protocols to the follow-up sessions.

A single B-scan centered at the fovea from both the pre-injection and post-injection OCT images was used. Contrast and brightness were adjusted to ensure optimal demarcation of image features, where optimal visualization of the ellipsoid zone was the main indication for optimal image quality. Care was taken to ensure that both the pre-injection and post-injection OCT images were overlapping and centered at the fovea. This was done using the enface scout image provided by the machine’s built-in software. Cross-sectional images obtained and centered at the center of the foveola were exported. 

The Fiji software was used for image processing before image segmentation [11]. The images were cropped to have 6 mm horizontal and 5.5 mm vertical images with the fovea being the center. The 6 mm width was obtained by first calibrating the pixel to micro-meter scale according to the scale provided by the OCT image, followed by the cropping function. After cropping the OCT section, no machine tags were present on the cropped image. The 6 mm width will cover the central foveal region, along with the inner and outer macular area [12,13,14]. The Computer Vision Annotation Tool (CVAT) platform for image segmentation [15].

### 2.3. Image Annotation and Classification

Each pre-injection image annotation was performed by a professor-degree retina specialist along with two ophthalmologists trained on OCT image interpretation and the image annotation software. After extensive literature review to determine important OCT image-based features that were related to response to anti-VEGF and overall prognosis, as well as the ability to annotate such features on images, we found five main features to be annotated:

- Inner intra-retinal fluids: Cystic fluid between inner limiting membrane (ILM) and retinal pigment epithelium (RPE), and within the inner nuclear and plexiform layers [16].
- Outer intra-retinal fluids: Cystic fluid between ILM and RPE, and within the outer nuclear and plexiform layers [16].
- Sub-retinal fluid: Hypo-reflective area between the RPE line and ellipsoid zone [16].
- Hyper-reflective foci: small discrete, well-circumscribed, dot-shaped lesion, with equal or greater reflectivity than the RPE band and without back-shadowing [17,18].
- Disruption of ellipsoid zone (EZ), described as the second hyper-reflective line after RPE [19].

Table 1 details the five main features and their corresponding RGB mask color values on the annotation software. 

**Figure 1:**
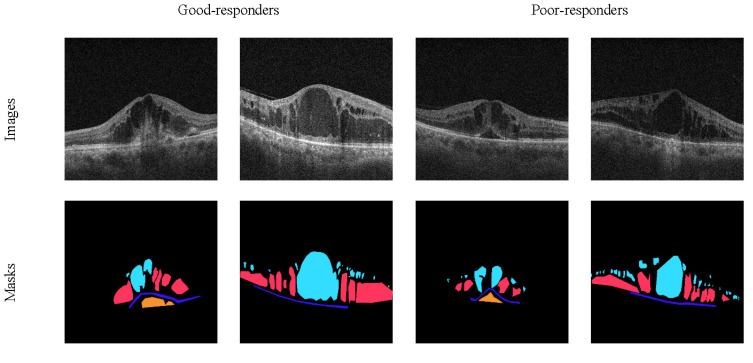
Some instances of the dataset, where the input images are shown in the first row and corresponding annotations are depicted in the second row. The first two samples belong to patients with good responses while the next two samples show the poor responses to the anti-VEGF treatment.

Images were classified based on the change in central OCT foveal thickness before and after intravitreal anti-VEGF injection, where good response on OCT was defined as reduction of thickness by >25% or 50 µm by 3 months [20]. Figure 1 shows samples of our dataset and corresponding segmentation mask.

### 2.4. Data Preparation

Our data preparation stage follows the offline sample-wise normalization and resizing process. To this end, each sample was resized to 512 × 512 pixels. Then for each sample, the image mean was subtracted and divided by the standard deviation to reduce the effect of the intensity range. Furthermore, for the training data, the data augmentation method with horizontal flip and small intensity shift was randomly performed.

### 2.5. Segmentation Model

**Figure 2:**
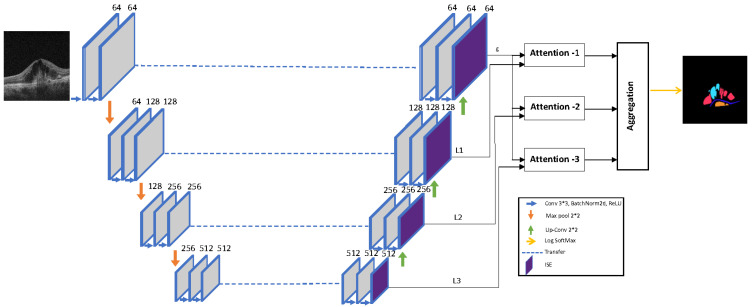
Overview of the proposed segmentation model’s architecture. The Inception-Squeeze-Excitation (ISE) module included in the decoding path to extract hierarchical semantic representation. Furthermore, the multi-level attention mechanism is utilized to extract multi-scale representation.

The segmentation model was based on the U-Net structure. The U-Net, a convolutional network for biomedical image segmentation, is a symmetrical model which is well designed to capture both semantic and high-resolution information [21]. Even though the U-Net model can capture local information, the structure is not completely designed for region-sensitive inference. In other words, to retrieve the diabetic sign from the DME not only the local representation is important but also the entropy of the area. To model such region-sensitive representation, squeeze excitation layers were first included into a decoding path of the model to formulate the region sensitivity then the convolutional blocks were reformed into an inception module to extract hierarchical semantic information. The resulted representation can describe regions based on both local variation and semantic information. The proposed architecture is shown in Figure 2. 

To formulate the segmentation model, the sample $X^{H^{\prime }\times W^{\prime }\times C^{\prime }}$ was considered as an input to the model. Where *H* and *W* are the spatial dimensions and *C* is the number of channels. The network initially applies the encoder block (parametrized with $f_{\theta }$) to encode the input image into a low dimensional latent space ($F^{H\times W\times C}$).

(1) F=f(X;θ)

In the next subsections, the inception, squeeze and excitation, and attention layers are discussed.

### 2.6. Feature Re-Calibration Module 

Although the feature vector generated from the encoder network contains high-level semantic information, it was unable to encode the interdependencies feature among the channels. Squeeze-and-Excitation (SE) module introduced a building block for CNNs that improved channel interdependencies at almost no computational cost [22]. The SE module at first, calculates the importance of each channel using the global information. Then using the extracted global information, it learns the scale parameters (parameters *W*_1_ and *W*_2_) for each channel based on how informative they are.

(2) $$GAP^{C}=\frac{1}{H\times W}\;\overset{H}{\underset{i}{\sum }}\overset{W}{\underset{j}{\sum }}F^{C\;}\left(i,j\right)\;$$

(3) $$w_{c}=\sigma \left(W_{2}\delta \left(W_{1}GAP^{c}\right)\right)$$

Finally using the scale parameters, the re-calibrated representation $F^{\prime }$ was produced.

(4) $$F^{\prime }=W_{c\;}F\;$$

### 2.7. Inception Layer

**Figure 3:**
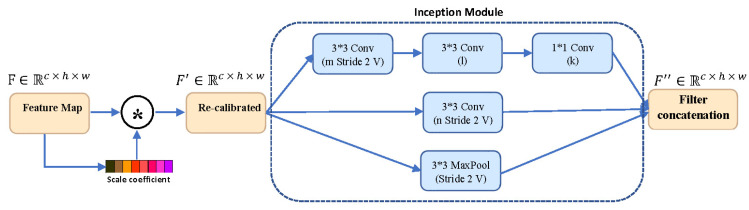
Our Inception Squeeze Excitation (ISE) block. This module scales the encoded feature F and then utilizes the inception module to generate transformed feature map.

To extract the hierarchical representation, we modified the last convolution layer of the decoding path with the inception module. The main concept of the inception architecture was to deploy multiple convolutions layers with different receptive field sizes, in parallel, to capture multi-scale representation [23]. To do so, the inception module on the output of SE module was utilized.

(5) $$F^{\prime \prime }=↓\left(F^{\prime }\right)\;\pm \;↓\left(\delta \left(K_{1}*F^{\prime }\right)\right)\;||↓\left(\delta \left(K_{3}*\;\delta \left(K_{2}*F^{\prime }\right)\right)\right)$$

where ↓ shows the down-sampling operation, ± indicates concatenation operation, and δ refers to the ReLU activation function. Figure 3 shows the Inception Squeeze Excitation (ISE) block architecture.

### 2.8. Multi-Scale Attention

Describing an object of interest in a multi-scale fashion not only produces a rich representation but also a set of scale-independent descriptions. To further enhance this representation, the attention mechanism on top of the multi-scale representation was included. The objective of the attention layer is to highlight the importance of each activated feature map with respect to the object of interest [24]. The attention mechanism is visualized in Figure 2.

### 2.9. Model Training

The segmentation model was trained using the combination of focal loss and dice coefficient (loss). The focal loss is an extended version of cross-entropy loss which is designed to address the class imbalance during training. This loss function automatically down-weights the contribution of easy examples during training and rapidly focuses the model on hard examples. Dice Coefficient, on the other hand, is a statistical tool that measures the similarity between two sets of data. These two losses were combined to train the segmentation model. It is worthwhile mentioning that for focal loss parameters, ‘gamma = 2’, and ‘alpha = 0.25’ were used and these worked well in the experiments. The whole network was trained using the RMSprop optimizer with batch-size 2 for 200 epochs. The RMSprop optimizer avoids both the vanishing and exploding gradient problems. A learning rate of 1 × 10^−4^ was used which was reduced by a factor of 0.5 when there is 10 successive non-progress on the validation loss.

### 2.10. Classification Model

**Figure 4:**
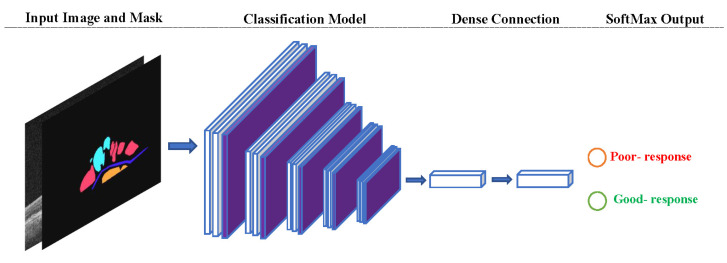
Classification Model Architecture. The classification model receives the predicted mask alongside the input image to perform initial attention mechanism.

In the classification stage, several well-known classification models pre-trained on image-net weights were used. To this end, a series of EfficientNet networks [25], VGG [26], ResNet [27], and DenseNet models were used [28]. The inputs for the classification network were both, the original OCT image of the patient and the segmented image from the segmentation part (Figure 4). This will enable the network to focus on important areas and ignore the parts related to the background image. The classification network was trained for 200 epochs using the Adam optimization with learning rate 1 × 10^−4^ and batch-size 2.

### 2.11. Performance Measures

To perform the evaluation of the proposed method on the test set, several well-known metrics were utilized. The terminologies used to describe how metrics are calculated are given below. 

True-Positive (*TP*) refers to the predicted label that is correctly predicted as a diabetic class. 

False-Positive (*FP*) refers to the predicted label that is falsely predicted as a diabetic class.

True-Negative (*TN*) refers to the predicted label that is truly labelled as background pixel. 

True-Negative (*TN*) refers to the predicted label that is falsely labelled as background pixel.

Area Under the Curve (*AUC*) represents the degree of separability, which is useful when the objective is to demonstrate the effectiveness of the model for separating *TP* and *FP* rate.

Accuracy shows the percentage of correct prediction,

(6) $$ACC=\frac{TP+TN}{TP+TN+FP+FN}$$

Specificity measures the proportion of *FP* that are correctly identified by model,

(7) $$Specificity=\frac{TN}{TN+FP}$$

Sensitivity measures the proportion of predicted *TP* that are correctly identified by model,

(8) $$Sensitivity\left(Recall\right)=\frac{TP}{TP+FN}\;$$

Precision measures the proportion of *TP* against all *T* predictions,

(9) $$Precision=\frac{TP}{TP+FP}\;$$

*F*1 score, also known as balanced *F*-score or *F*-measure, is a weighted average of the precision and recall,

(10) $$F1=\frac{2*\left(Precision*Recall\right)}{Precisio+Recall}\;$$

### 2.12. Real World Testing

To compare the model’s response prediction accuracy with practicing general ophthalmologists, pre-treatment macular OCTs for five patients who satisfied the inclusion criteria specified above were collected but were treated after the development of the model (i.e., not previously included in the model training stage). The proposed model was evaluated on these images as well to find the number of correctly classified images (i.e., accuracy). A Google form-based survey containing the same five pre-treatment macular OCTs was then distributed to ophthalmologists and trainees to compare their classification accuracy with the developed model. We targeted junior and senior residents, general ophthalmologists, and retina specialists from ophthalmology departments at The University of Jordan, Royal Medical Services, ministry of health, and private sector. Heads of ophthalmology departments and centers were contacted to distribute the survey. The form started by defining the nature of the study and the definition of good and poor responses, followed by a consent to participate, the level of experience, and finally the five macular OCTs with choices being “Good response, “Poor response”, and “Don’t know”. The outcome of this comparison is discussed in the Results section. 

### 2.13. Implementation Settings and Statistical Analysis 

The proposed models were implemented using PyTorch version 1.8.0 [29], with NVIDIA CUDA version 10.0 (Santa Clara, CA, USA), Single GPU NVIDIA TITAN X and 64 GB RAM. For our algorithm approximately 6 GB should be enough depending on the batch size. The models were trained and evaluated on Linux (Ubuntu) operating system (also compatible with Windows) and were coded in Python 3.9. The performance measurement and statistical analysis were performed using the publicly available libraries including Scikit-learn version 0.20.3.

## 3. Results 

A total of 101 patients eventually met the inclusion criteria set out, and their pre-treatment images were annotated, then their class was determined by the clinical team (i.e., general ophthalmologists and retina specialist). None of the included images were excluded by the computer science team after inclusion by the clinical team. The mean age was 63.34 (10.11) years, and 63 (62.4%) were men and 38 (37.6%) were women. Of the 101 patients, 60 patients were labeled as good responders to treatment (i.e., positive to treatment), and 41 patients were labelled as poor responders to treatment (i.e., negative to treatment). Table 2 details the characteristics of the included sample. 

The described segmentation deep learning method resulted in segmentation accuracy of 95.9%, an AUC of 93.4%, a specificity of 98.9%, a sensitivity of 87.9%, a precision of 80.7%, an F1 score of 83.9%, and a dice score also of 83.9%. To further scrutinize the effect of each module added to the baseline U-net model, the performance of the model was analyzed after each additional layer was added starting from the baseline model (U-Net). Table 3 details the overall result of the segmentation network from baseline U-net model to our proposed model, along with each layer addition in between, and shows how our proposed method outperformed all other methods. 

**Figure 5:**
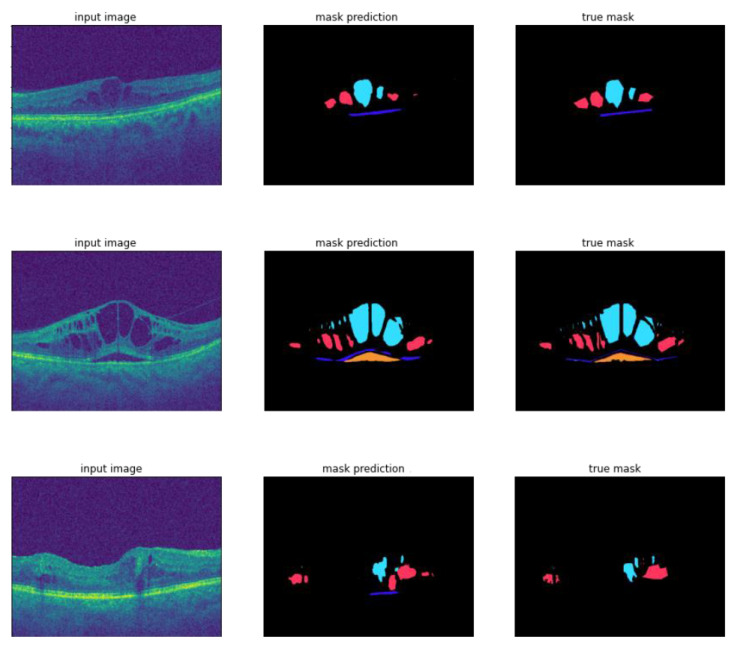
Sample of segmentation results including the input image, mask prediction and true mask.

In Figure 5, samples of the segmentation results are provided and show how the model accurately segmented DME regions. 

Regarding classifying patients’ images into good and poor responders, we found that the best performing network was the EfficientNet-B3 network, with classification accuracy reaching 75%. Using this network, we evaluated two different settings where in the first setting we used only the image itself as an input while in the second strategy we included the predicted mask as an extra channel to the input image. The results show that including the predicted mask in the input layer not only performs as an initial attention mechanism but also improves the generalization performance. In Table 4, the comparison results are detailed.

**Figure 6:**
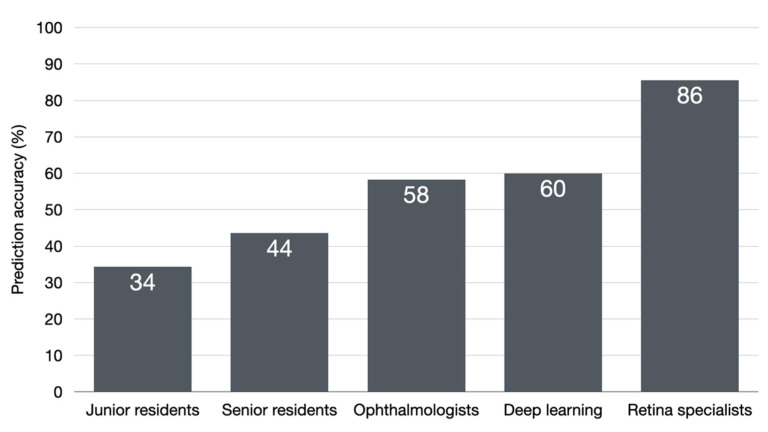
Classification accuracy of the deep learning model compared to different levels of ophthalmology trainees and specialists.

On real world testing of the developed model on newly collected macular OCTs, the model was able to correctly classify three out of five images into good or poor responders (60% accuracy). In regard to surveyed ophthalmologists and trainees, a total of 34 participants completed the survey. They were seven junior ophthalmology residents, nine senior ophthalmology residents, 13 general ophthalmologists, and 5 retina specialists. Figure 6 shows classification accuracy of the proposed deep learning model compared to different levels of ophthalmology trainees and specialists. The deep learning model achieved an accuracy higher than junior (34%) and senior (43.6%) ophthalmology residents, comparable to general ophthalmologists (58.3%), but still lower than retina specialists (86.3%).

## 4. Discussion

With the rise of machine and deep learning methods and their rapid advancement, there has been recent interest regarding predicting response to anti-VEGF from baseline OCT among diabetic patients with DME. To our knowledge, only two previous works tried to use deep learning to predict response to anti-VEGF from baseline OCT among patients with DME. The first is the work by Rasti et al. where they developed a deep learning model using CADNet as a baseline model, followed by adding certain convolutional layers to improve model accuracy [30]. They performed their experiment using baseline OCT images of 127 patients. They showed that using relatively small sample size, the model was able to predict and classify patients with high accuracy. The other similar work is by Cao et al. where they used separate deep learning models to be trained on different features, including hyper-reflective foci, intra-retinal, and subretinal fluid, which might lead to higher computational demand than feature extraction using one model [31]. After that, they developed a machine learning model to predict treatment outcomes, an approach that is considered inferior and less sophisticated compared to deep learning [32]. Despite the limitations in Cao et al. study, their strength was including relatively large sample size. Regarding model accuracy, it is difficult to compare models that were not tested on similar data, which is the case for the mentioned studies and ours. While there are plenty of openly accessible datasets of medical and ophthalmic imaging, they are usually cross-sectionally collected data [33]. In the case of predicting response to treatment, longitudinal openly accessible data with strict inclusion and exclusion criteria need to be considered, which is not currently available.

There are several important advantages of developing a model that can predict response to anti-VEGF before starting treatment. A recent review article proposed that AI-based tools could be applied to generate patient-customized prognostic data and predict individual treatment needs, reducing the time needed to optimize a patient’s treatment regimen [34]. This is especially important for low-resource settings, where patients themselves may not be able to afford treatment, and for counseling where a prediction about their good or poor potential response may aid in their decision making, considering the burden and cost of anti-VEGF treatment [35]. Moreover, the recent 2019 Coronavirus pandemic, which led to enormous pressure on health systems also showed the importance of such a model in triaging patients toward the limited number of available resources [6].

There are several novelties in the work presented here. Including five different features of DME on OCT and accurately segmenting them has not been previously performed. Moreover, the approach of having two deep learning models, where the output of the first was used as an input to the second has not also been done before for DME treatment response prediction using OCT. In our design, a cascaded structure was followed. In the first step, the segmentation model to generate the segmentation map for the input image was applied. Then in the second step, the classification phase on the input image was performed by considering the predicted mask. More precisely, the predicted mask was included in the input layer of the classification model to perform the initial attention. This supervisory signal not only helps the model to focus on the more informative area but also diminishes the effect of background on the inference process. Another advantage of the current study is that it was based on data from Jordan, with patients from Middle Eastern ethnicity. Developing models on a wide variety of populations is essential to develop highly accurate models from small datasets using transfer learning [17,36]. Another point to consider here is that the accuracy of predicting response to treatment did not improve upon including patients’ characteristics, including age, gender, and other comorbidities. The reason behind this finding can be explained by previous deep learning projects on ocular imaging, which showed that deep learning models can already predict such characteristics from ocular imaging [37,38]. 

Several limitations still need to be considered upon interpreting the results of the current study. The main limitation is the small sample size that was used to train the models, which was extracted from a single OCT machine type. To have a more reliable model that can be utilized in a real-life setting, a larger sample size from different machines needs to be considered. Another aspect to be considered is the use of an anatomical outcome to determine the response to anti-VEGF, an approach commonly followed and advocated by several previous landmark trials [39,40,41,42]. However, the use of a functional outcome (e.g., visual acuity) to assess the response to anti-VEGF might result in a more patient preferable result.

## 5. Conclusions

A DL model was developed to segment macular OCT for DME patients, and its output was then fed into another DL model that classifies the patients based on their pre-treatment macular OCT into good or poor responders to anti-VEGF treatment. The model showed a high accurate segmentation and an acceptable accuracy for classification, despite the small number of training data. This is the first deep learning project that can predict response to anti-VEGF based solely on pre-treatment OCT. Despite that, there is still a long way to go before implementing such a model in clinical practice, a model that is expected to improve resource allocation, triaging and counseling patients who would benefit most from treatment and have adverse outcomes if treatment was delayed. It would also improve choosing different patients in clinical trials, where poor responder patients might be further investigated for better understanding. 

## Figures and Tables

**Table 1:** Annotation classes and corresponding masks RGB values for our dataset.

Classes	RGB Colour	RGB Value	Class Number
Background	Black	0,0,0	0
Inner Intraretinal fluid	Soft Blue	51,221,255	1
Disrupted ellipsoid zone	Strong Blue	53,15,247	2
Sub-retinal fluid	Orange	245,147,49	3
Hyper-reflective dot	Yellow	250,250,55	4
Outer intra-retinal fluid	Red	255,53,94	5

**Table 2:** Clinical characteristics of included sample.

		Mean	Standard Deviation	Count	Column N %
Age (years)		63.34	10.11		
Gender	Female			38	37.6%
	Male			63	62.4%
Eye laterality	Left			44	43.6%
	Right			57	56.4%
Severity of DR	Mild non-proliferative diabetic retinopathy			12	11.90%
	Moderate non-proliferative diabetic retinopathy			28	27.70%
	Severe non-proliferative diabetic retinopathy			19	18.80%
	Proliferative diabetic retinopathy			42	41.60%
Central macular thickness pre-treatment (μm)		475	146		
Central macular thickness post-treatment (μm)		382	149		
Best corrected visual acuity pre-treatment		0.258	0.205		
Best corrected visual acuity post-treatment		0.334	0.211		
Functional outcome	Worsened			7	
	Stable			57	
	Improved			37	
Prior history of argon laser	No			58	
	Yes			43	
Prior history of anti-VEGF	No			38	
	Yes			63	
Prior steroid injections				4	
Phakic status	Phakic			69	68.3%
	Pseudo-phakic			32	31.7%

**Table 3:** Performance comparison on DME dataset for different approaches.

Methods	AUC	Accuracy	Specificity	Sensitivity	Precision	F1 Score	Dice Score
Baseline (U-Net)	0.904	0.925	0.973	0.836	0.772	0.802	0.802
U-Net + SE	0.912	0.937	0.981	0.844	0.786	0.812	0.812
U-Net + ISE	0.921	0.951	0.985	0.846	0.788	0.817	0.817
Proposed Method (U-Net+ ISE+Attention)	0.934	0.959	0.989	0.879	0.807	0.839	0.839

**Table 4:** Classification performance of different models for predicting the effectiveness of anti-VEGF treatment.

Methods	Accuracy %	Precision %	F1 Score %	AUC	Sensitivity	Specificity
VGG	65	60	70	70.93	70.77	76
ResNet	70	65	75	76	75.82	78
DenseNet	70	65	75	76.01	75.82	78
EfficientNet-B3 (image)	70	65	75	76.03	75.84	78
EfficientNet-B3 (image + mask)	75	70	80	81.07	80.88	84

## Data Availability

Data used in this study is available with the corresponding author upon reasonable request.
